# Don’t Forget Rare Causes of Postpartum Headache! Cases Report and Literature Review

**DOI:** 10.3390/medicina57040376

**Published:** 2021-04-13

**Authors:** Marco Di Paolo, Aniello Maiese, Ornella Mangiacasale, Barbara Pesetti, Simone Pierotti, Alice Chiara Manetti, Massimiliano dell’Aquila, Angela De Filippis, Emanuela Turillazzi

**Affiliations:** 1 Section of Legal Medicine, S. Chiara Hospital, University of Pisa, via Roma 55, 56126 Pisa, Italy; marco.dipaolo@unipi.it (M.D.P.); simone.pierotti@libero.it (S.P.); alicechiara812@gmail.com (A.C.M.); emanuela.turillazzi@unipi.it (E.T.); 2 Section of Anesthesiology, Department of Surgical, Medical, Molecular Pathology and of Critical Area, University of Pisa, 56126 Pisa, Italy; ornellamangia87@gmail.com; 3 Section of Maternal and Child Anesthesia and Resuscitation and Santa Chiara, Department of Anesthesia and Resuscitation, Azienda Ospedaliero Universitaria Pisana, 56126 Pisa, Italy; bafpesetti@inwind.it; 4 Department of Anatomical, Histological, Forensic and Orthopaedic Sciences, Sapienza University of Rome, Viale Regina Elena 336, 00161 Rome, Italy; massimiliano.dellaquila@uniroma1.it; 5 Fetal Medicine Unit, Di Venere Hospital, 70121 Bari, Italy; angela.defilippis@hotmail.it

**Keywords:** post-dural puncture, magnetic resonance imaging, brain, pre-eclampsia/eclampsia, migraines

## Abstract

Headache is a common finding in the postpartum period, caused by a spectrum of different conditions. Most headaches in the postpartum period are self-limiting and benign in etiology, but there are some potentially serious causes to be considered. We disclose two cases of postpartum headache, initially considered as post-dural puncture headache (PDPH), that evolved into a harmful condition and showed that an expanded differential diagnosis for headache in the postpartum is mandatory, requiring a high level of attention from health professionals. In fact, a careful examination of the medical history, physical examination, and the recognition for the need for early neuroradiological imaging should increase diagnostic accuracy.

## 1. Introduction

Postpartum headache is described as a complaint of headache and neck or shoulder pain in the first 6 weeks after delivery [1]. Postpartum headache is quite common: it has been estimated that up to 39–40% of women experience headaches in the postpartum weeks [2,3]. Tension (39%), pre-eclampsia/eclampsia (24%), migraines (11%), hemorrhage, thrombosis, or vasculopathy (10%), and post-dural puncture headache (PDPH) (16%) are the reported possible causes of headaches in the postpartum [2,4,5,6,7,8].

Because of the multiple potential etiologies and complications that may evolve, the identification of potentially harmful etiologies is of paramount importance for clinicians who need to quickly exclude or confirm potentially life-threatening diagnoses [9].

Here, we present two obstetric cases in which a diagnosis of PDPH was initially made. However, due to underlying cerebrovascular disorders, the neurological conditions of the patients deteriorated, leading to further investigations. In both cases, patient made a claim against the physicians and health care institution.

### 1.1. Case Report 1

A 37-year-old woman, gravida 1-para-0 (BMI = 28), with no significant past neurological or medical history, was hospitalized with a diagnosis of premature rupture of membranes in pregnancy at 39 + 3 weeks of gestation. The patient, after signing informed consent, underwent three attempts of epidural analgesia with a Tuohy needle of 18 Gauge. Suspecting accidental puncture of dura mater, they modified the anesthetic technique and started intravenous administration of Remifentanil. Moreover, induction of labor was carried out, with vaginal delivery after 1 h and 15 min. After 10 h from the initial attempt of epidural anesthesia, the patient begins to complain of symptoms as orthostatic headache and irradiated neck pain. The pain increases progressively, affecting the entire rachis. Consequently, she received treatment with crystalloids (lactated Ringer) in continuous infusion and painkillers (paracetamol), also during the 1st postpartum day.

On the second day postpartum, infusion therapy was resumed 12 h after the release of peripheral venous cannula, with substitution of paracetamol with caffeine orally, without apparent benefit. The headache started to appear also in supine position.

On the third day postpartum, painful symptoms worsened, with the appearance of vomiting and extreme physical prostration; hemodynamic parameters within the normal limits. On the same day, an epileptic generalized tonic–clonic seizure occurred: an electroencephalogram and an encephalon-skull-CT were performed, but no diagnostic elements emerged.

On the fourth day postpartum, a second convulsive episode appeared, following which the patient underwent a cranial encephalic MR, which demonstrated a reduction in the size of periencephalic liquor spaces at the cranial base and in the posterior cranial fossa, associated with caudal dislocation of the encephalic trunk, dilatation of dural venous sinuses, and contrast impregnation of the dura mater, findings compatible with vascular hypotension and bilateral frontal cortical infarctions with bilateral right and temporal dominance for venous stasis thrombosis in relation to the caudal shift of the brain. Consequently, the patient was transferred to the Stroke Unit, where she was treated with oral anticoagulant therapy, then replaced with low molecular weight heparin and antiepileptic therapy (levetiracetam i.v.) and she underwent blood tests, which showed leukocytosis, anemia, and thrombocytosis, in absence of a hereditary thrombophilic state. During the hospitalization, the patient maintains a negative neurological objectivity and reports an improvement of the headache, documented by the NMR images, until disappearance, 10 days after dural puncture.

Two months later, a third MRI survey showed a resolution of the picture, without alterations of the brain signal or abnormal impregnations in intra and extra axial, with liquor spaces within the norm. From a clinical point of view, there were significant memory changes and an anxious depressive state, in pharmacological and psychotherapeutic treatment, for which compensation has been received for iatrogenic damage.

### 1.2. Case Report 2

A 32-year-old, healthy, gravida 1-para 0 woman (BMI = 24.5), with no significant past neurological or medical history, hospitalized for premature rupture of membranes in pregnancy at 40 weeks + 3, after signing informed consent, underwent epidural analgesia during which the dura mater was perforated. A few hours after the puncture, she complained of postural headache, treated with intravenous hydration with crystalloids and caffeine, which continued until the fourth day.

On the fifth postpartum day, left facio-brachio-cruralplegia appeared, associated with ipsilateral brachio-crural hypoesthesia. Despite the persistence of the symptoms, the therapy was unchanged and no diagnostic intervention was undertaken, until an episode of loss of consciousness associated with convulsive tonic-clonic manifestations occurred, at normal hemodynamic parameters. The patient was transferred to the Intensive Care, underwent an encephalic MRI, which showed findings compatible with posterior reversible encephalopathy, and an electroencephalogram, which demonstrated right frontal-temporal crisis, for which treatment was undertaken with magnesium sulphate, corticosteroids, thiamine, low molecular weight heparin, and benzodiazepines.

On the eighth postpartum day, a second seizure crisis occurred: a further encephalic MRI was performed, showing ischemic hemorrhagic evolution of encephalopathy, for which mannitol therapy was added. Subsequently, the patient was transferred to the Neurology department to carry on the therapy. During the hospitalization, episodes of pulsating frontal headache and a vertiginous syndrome appeared, with an unchanged instrumental picture of RMN and CT.

After discharge, the patient underwent therapy with amitriptyline, nimodipine, and phenytoin, and even though she underwent prolonged rehabilitative treatment to recover the motor deficit, hypotonia in the upper left hand, left hemisomatic hyposthenia, and related episodes of headache and tinnitus remained.

## 2. Discussion

Headache is frequent following childbirth [10], regardless of the history of previous headaches [4,11,12,13,14]. Evidence exists that the puerperium is a very vulnerable period for the development of several primary and secondary headache disorders, mainly due to hormonal, physiological, procedural, and psychological factors [15]. Consequently, the spectrum of differential diagnosis for acute, postpartum headache is broad. Primary headache disorders, tension type and migraine, are reported as the most common headache in pregnancy and puerperium [16,17,18], accounting for approximately 50–75% of headaches [19]. The most frequent secondary causes of postpartum headache are represented in Table 1.

If diagnosis is not promptly achieved and appropriate treatments are not started, many of these conditions can increase maternal morbidity or mortality. It is noteworthy that if these young, usually previously healthy, women have a poor outcome, the clinical, social, and medico-legal impact is often significant [15]. However, as the features of many types of postpartum headaches may overlap among themselves and with those of migraine [19], the differential diagnosis between primary and secondary headache disorders may be challenging, particularly for the most severe patients referred for urgent neurological consultation, and when atypical symptoms are present [16,24].

In a recent single-center retrospective study of consecutive postpartum women presenting with acute headache and receiving neurological consultation, Vgontzas and Robbins reported that secondary headache is more frequent during the postpartum period [25]. On the same line, Klein et al. highlighted that, due to their high incidence, primary headaches and PDPH are the most immediate diagnostic hypotheses in women who complain of postpartum headache, especially considering the fact that many women receive epidural analgesia during labor [19].

The treatment of choice for PDPH is bed rest, analgesia, intravenous hydration, and caffeine supplementation. Patients who do not respond to this treatment within 48 h may require a blood patch. The treatment of secondary causes of headache in the postpartum period often requires collaboration with consulting services both for acute management and risk factor modification [26].

Currently, it seems to be unknown whether there is a significant association between post-dural puncture headache and such a rare complication as subsequent intracranial subdural hematoma. Moore et al., in their cohort study, showed that the presence of post-dural puncture headache after neuraxial anesthesia in childbirth during labor, compared with no headache, was associated with a small but statistically significant absolute increase in the risk of intracranial subdural hematoma, but further studies are needed to ascertain whether there is a causal [27]. Moreover, Algahtani et al. reported a case of adverse events of epidural analgesia using the loss of resistance to air technique. They believe that this technique should be studied further in both human and animal models in regards to complications, particularly pneumocephalus. At least, the patient should be informed about this potential complication if this technique is used. This technique is associated with more complications if air is used to identify the epidural space instead of normal saline [28].

The occurrence of pneumocephalus after epidural analgesia is rare with only a few cases reported in literature [29]. Post-dural puncture headache is the most common complication of unintended dural puncture with an epidural needle. Pneumocephalus and the following headache can be differentiated from PDPH by differences in clinical presentation. PDPH usually occurs within 1 to 4 days of the inadvertent dural puncture and continues for approximately 4 days; it is characterized by a reduction of the headache in the supine position. The headache in pneumocephalus usually occurs immediately following the procedure and continues to deteriorate in spite of any postural changes and persists even in the supine position [30]. Diagnosis of pneumocephalus is confirmed by a brain CT, which has the ability to detect as little as 0.5 ml of intracranial air [29].

One study showed that most diagnoses after neurological counselling were secondary headaches, and PDPH was the most common, followed by postpartum preeclampsia and then other vascular disorders [25]. Burch recommends that in women showing nagging postpartum headaches, secondary causes should absolutely be taken in account. Furthermore, she highlighted that in almost all cases of suspected secondary headache, imaging of the brain and blood vessels is necessary [31].

Hiremath et al. analyzed 40 patients with peripartum headache and seizures, which underwent cross sectional imaging with CT and MRI in order to evaluate a range of causes and their typical findings on this imaging techniques. Eclamptic encephalopathy and cortical venous thrombosis were the main causes, and they concluded that rational use of CT and MRI in the early appearance of the symptoms helps in characterizing the lesion and providing the proper treatment [32,33].

Until now, limited studies have been published on the use of MRI and the typical findings in patients with postpartum headache [34,35]. In a recent cross-sectional study, Shobeiri and Torabinejad showed that in a consistent number of patients with postpartum headache, abnormal brain MRI findings were present. Furthermore, both onset of postpartum headache in the first 5 days after delivery and appearance of seizures were considered as significant predictors of an abnormal brain MRI [36]. MRI scans can be a useful tool for the proper diagnosis of postpartum head-ache, which is usually reached in delay due to headache being a common symptom, and it is initially confused with eclampsia. Prompt diagnosis of these conditions and proper treatment improves outcome and prognosis [37]

Katsevman et al. presented a case of severe posterior reversible encephalopathy syndrome (PRES) associated with recent pregnancy and eventual diagnosis of eclampsia, which required a decompressive suboccipital craniectomy. PRES is a neurotoxic state characterized by subcortical vasogenic edema within the brain causing neurologic sequelae. They showed that, due to confusing clinical and imaging data, a diagnosis of PRES is not always easily reached. This uncommon syndrome is potentially harmful if not recognized and treated properly. It may be associated with pregnancy, eclampsia/preeclampsia, and/or CSF drainage. Conservative management may be inappropriate and a craniectomy for decompression can be required even if intracranial hemorrhage is not present. [38].

Elie Sader and Melissa Rayhill, in their review, underlined that the epidemiology of headaches during pregnancy and the postpartum period is very different from that in males or nonpregnant females, partly due to the hemodynamic and hematologic changes which take place during pregnancy. The postpartum is a period characterized by a hypercoagulable state. Management of headache during pregnancy and lactation is largely reliant on a risk/benefit equilibrium, as many medications can produce adverse effects in the newborn [39].

As shown from the literature, a large number of cases of postpartum headaches are not recognized, and consequently, are slightly or not treated, often coming back after discharge for a revaluation [40]. Yilmaz and Çevik made a review to analyze the proper approach to headache and the strategies to promote best practice in pregnancy and lactation [41].

Emma Stanhope et al. [9], in order to establish a standardized approach for the management of patients with postpartum headache, tried to introduce a specific diagnostic tool, the PARTUM mnemonic: Pressure (blood pressure for pre-eclampsia/eclampsia), Anesthetic (post-dural puncture headache), Reversible (vasoconstriction syndrome), Thrombosis (cerebral venous sinus thrombosis, ischemic stroke), Use your brain (there are so many other causes of headache), and Migraine, which was first proposed by Lim et al. [10]. The aim of this tool is to enable clinicians to identify the cause of postpartum headaches faster and to allow life threatening diagnoses to be quickly excluded [9].

De Pietri et al. proposed to change the women’s management after delivery, increasing their participation in the diagnostic process of possible neuraxial analgesia complications. They started to give them a clear description of the potential complications related to dural puncture through the use of a leaflet with the necessary information and asking them to complete a questionnaire if they got a headache after discharge [42].

Our cases confirm that, in women who underwent labor analgesia presenting with headache, PDPH may precede the missed diagnoses, due both to the anamnestic datum of labor analgesia, and to the clinical initial symptoms mimicking PDPH. A more rapid and critical assessment of the patient’s change in symptoms (as reported in our second case) could trigger a timelier diagnostic process (through the use of MRI and CT), and consequently, shorten the timing of the diagnosis and appropriate management. This could be obtained both through the use of a standardized approach, as suggested with the diagnostic tool of PARTUM mnemonic, and through the use of tools as questionnaire and leaflets, which should increase women’s awareness and their active participation in the diagnostic process.

From a medicolegal point of view, informed consent is a cornerstone in order to explain to the patients that secondary headache are conditions not avoidable despite an early diagnosis and a proper management of symptoms, and that there is no scientific evidence of the causal role of PDPH in their onset.

## 3. Conclusions

Headache is a frequent complication of the postpartum period, caused by different conditions, both benign and life threatening. Although progress has been made in the field of obstetric care in the last decades, currently, there is no scientific evidence to completely avoid the possibility of postpartum headache onset. Anesthesiologists play a key role: they are often the first physician called to review the patient, and thus, they need to keep in mind all possible causes, even rare, of postpartum headache, to deal with them in order to establish early and appropriate management and limit consequences for the patient and medicolegal proceedings.

## Figures and Tables

**Table 1:** The differential causes of secondary postpartum headache, helpful elements for diagnosis, and common management. CSF: cerebral spinal fluid; EBP: epidural blood patch; NSAIDs: non-steroidal anti-inflammatory drugs; MRI: magnetic resonance imaging; CTI: computed tomography imaging [20].

Secondary Postpartum Headache Etiologies	Signs and Symptoms	Diagnosis	Treatment
Pre-eclampsia/Eclampsia	Hypertension, headache, and altered mental status until unconsciousness	Clinic with laboratory findings of thrombocytosis, possible alteration of liver function, proteinuria	Pharmacological: blood pressure control with labetalol and nifedipine, antiseizure with phenytoin, diazepam, midazolam, and Magnesium e.v.
PDPH	Postural headache worsening with activity, subsiding in 15 min with supine position after accidental dural puncture (ADP)	Clinical diagnosis of ADP during analgesia overflow of CSF from Touhy needle, or after positioning of epidural catheter for aspiration of CSF or anesthesia after injection of a test dose of anesthetic	If the epidural catheter is inserted, leave in place for 24 h. Keep antalgic position. Avoid dehydration with eventual e.v. supplementation. Pharmacologic: analgesics and NSAIDs Invasive treatment: EBP if pharmacologic treatment fails after 2 weeks [21]
Cerebral venous sinus thrombosis (CVST)	Aspecific headache with possible focal signs, loss of consciousness, and seizure	MRI	Pharmacological: control of seizures and anticoagulation therapy [22]
Subarachnoid Hemorrhage (more common in presence of MAV)	Sudden intense headache unilateral with nausea, neck stiffness, and loss of consciousness	CTI	Possible neurosurgery in selected case
Posterior reversible Encephalopathy syndrome (PRES)	The following can be present: hypertension, headache, vomiting, visual disturbance, altered mental status until unconsciousness, and seizures	CTI MRI	Pharmacologic: to control blood pressure, phenytoin, midazolam, or diazepam; to control seizure, corticosteroids for edema
Cerebral infarction/ischemia	Sudden headache, with vomiting, seizure, and possible focal deficit	Cerebral angiography	Specialist neurologic opinion for management
Meningitis	Fever, neck stiffness, and photophobia. Kernig and Brudzinski signs positive. Petechial rash possible	Lumbar puncture with examination and culture of CSF	Selected antibiotic therapy
Pituitary apoplexia (more common in presence of adenoma)	Retro-orbital headache and possible hormonal insufficiency (adrenocortical insufficiency, hypothyroidism) and diabetes insipidus	MRI and possible laboratory endocrinologic hormonal alteration	Correct hydro-electrolytic imbalance if present. Endocrinologic therapy to supply hormone deficiency. Possible neurosurgery in selected cases [23].

## Data Availability

Data available on request due to restrictions e.g., privacy or ethical reason.
